# Study on Electromagnetic Thermal Characteristics of Stacked REBCO Tapes Under Alternating Current with DC Bias

**DOI:** 10.3390/ma19101949

**Published:** 2026-05-09

**Authors:** Wei Chen, Yang Bai, Rong Jin, Fei Chi, Xinsheng Yang

**Affiliations:** 1 School of Electronic and Electrical Engineering, Wenzhou University of Technology, Wenzhou 325035, China; 20210386@wzut.edu.cn (W.C.);; 2 School of Data Science and Artificial Intelligence, Wenzhou University of Technology, Wenzhou 325035, China; 3 Key Laboratory of Magnetic Suspension Technology and Maglev Vehicle (Ministry of Education), Southwest Jiaotong University, Chengdu 610031, China

**Keywords:** REBCO tapes, DC bias, AC loss, electromagnetic–thermal coupling, stacked cable

## Abstract

In practical applications, high-temperature superconducting (HTS) cables or magnets may carry AC with DC bias, such as in superferric magnets, which can increase the AC loss of the cables or magnets. When the DC bias current is high, the resulting high loss can lead to a significant temperature rise in the cable or magnet and may even cause quench. Furthermore, different waveforms of the alternating current also result in different losses and temperature rises. Therefore, it is essential to investigate the AC loss of the cable under different current waveforms and DC bias levels using an electromagnetic–thermal coupling method. In this paper, an electromagnetic–thermal coupling model is used to investigate the AC loss and temperature rise characteristics of four stacked REBCO tapes under four typical current waveforms and various DC bias levels. The actual multilayer structure of REBCO tapes is considered in the numerical simulation, which facilitates the analysis of current distribution among different layers and its contribution to the total loss of the stacked cable. The results show that under zero DC bias or a small DC bias (0.1*I*_dc_), the square-wave current yields the largest AC loss, while the triangular-wave current results in the smallest AC loss. The losses generated by the sawtooth and sinusoidal currents are comparable and intermediate between those of the two aforementioned waveforms. When the DC bias current is moderate (0.5*I*_dc_) and the amplitude of the alternating current is greater than 0.5*I*_cable_, the loss of the cable increases rapidly. The loss generated by the square-wave current is the largest, followed by the sinusoidal current, while the sawtooth and triangular currents produce the smallest losses. When the DC bias current is high (0.9*I*_dc_), even a small amplitude alternating current results in high AC loss in the cable.

## 1. Introduction

REBCO tapes have significant application advantages in power systems, fusion reactor magnets, accelerator magnets, and magnetic resonance magnets due to their high current density, high critical field, and mechanical strength [1,2,3,4,5,6]. Among them, in synchronous accelerator magnets, transmission cables, magnetic energy storage systems, and fault current limiters, superconducting tapes operate under DC containing AC ripple components [7], which can cause losses and increase the temperature of the tapes. If the temperature rises too high, it may lead to tape quenching. Therefore, it is necessary to understand the losses and temperature rise caused by superconducting tapes under DC bias.

At present, a large number of studies have reported the AC losses of tapes, cables, and magnets under mixed AC/DC conditions and complex waveforms [7,8,9,10,11,12,13,14,15,16,17]. References [7,8,9,10,11,12] report the AC losses of HTS tapes, cables, and magnets under DC bias. A small DC bias has almost no effect on the AC loss of a single tape and stacked cables, while a large DC bias significantly increases the loss. References [13,14,15] reported the effect of harmonics on tape and coil losses, and the results showed that harmonic components have a significant impact on AC losses. References [16,17] report the influence of typical waveform currents on the AC losses of tapes and cables. The results indicate that under a certain amplitude of transport current, the square current waveform has the highest AC loss, while the triangular current waveform has the lowest AC loss. References [18,19,20,21] report the effects of more complex transmission currents and AC/DC background magnetic fields on the AC losses of high-temperature superconducting tapes and magnets. However, the above studies did not consider the impact of temperature rise caused by losses on the performance of the tape, and there are almost no reports on REBCO cable losses carrying typical alternating current waveforms under DC bias.

In this study, an electromagnetic–thermal coupling model was used to investigate the effects of different current waveforms and amplitudes on the AC losses and temperature rise in four stacked REBCO tapes under different DC biases. The four types of current waveforms are sawtooth wave, sine wave, square wave and triangular wave. The DC bias current is set to 0.1, 0.5, and 0.9 times the critical current of the cable, and the peak of the alternating current ranges from 0.1 to 0.9 times the critical current of the cable.

## 2. Numerical Model and Method

### 2.1. Geometric Models

**Figure 1:**
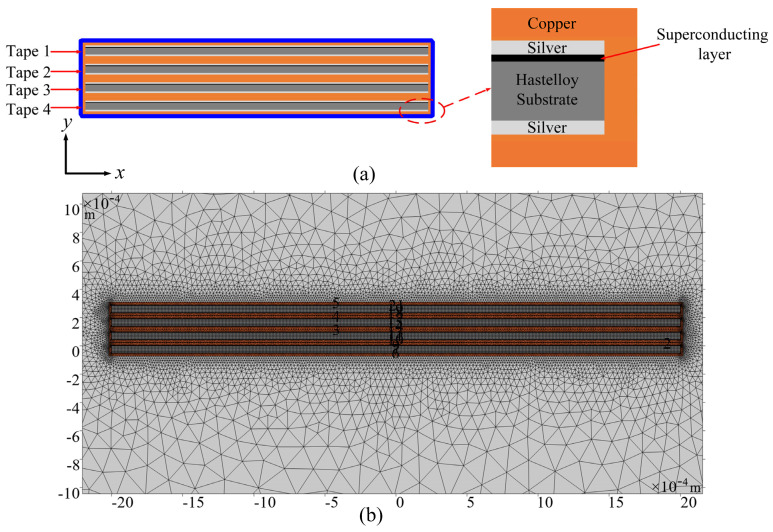
(**a**) A cross-sectional model of a cable consisting of four stacked REBCO tapes. (**b**) The meshing of the stacked cable.

**Figure 2:**
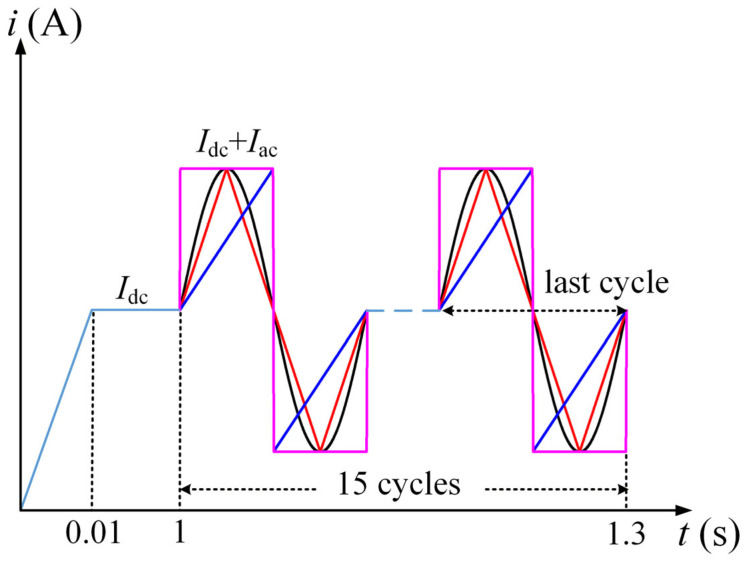
Alternating currents with different waveforms under DC bias. The black, red, pink and blue lines denote the sinusoidal, triangular, square and sawtooth alternating current waveforms superimposed on the constant DC bias *I*_dc_.

The geometric model of the REBCO cable is shown in Figure 1a. The cable is composed of four stacked REBCO tapes. Each REBCO tape consists of a copper stabilizer layer, a silver layer, a superconducting layer, and a Hastelloy substrate layer, based on the SCS4050 tape produced by Superpower [22]. The geometrical dimensions of the REBCO tape and the cable are listed in Table 1. The four superconducting tapes are numbered Tape 1, Tape 2, Tape 3, and Tape 4. Figure 1b shows the meshing of the stacked cable, where structured meshes are adopted for the superconducting layer, the silver layer and the Hastelloy substrate, while free triangular meshes are used for the copper stabilizer layer. Figure 2 shows the time-dependent waveform of the applied current for the cable, with the frequency of the alternating current being 50 Hz. The current is increased linearly from 0 A to the preset DC level within 0–0.01 s, held constant at *I*_dc_ during 0.01–1 s, and then subjected to 15 cycles of alternating current with varying amplitudes, superimposed on the constant DC bias, from 1 s to 1.3 s.

### 2.2. Numerical Method

The electromagnetic–thermal behavior of stacked REBCO cables is described by electromagnetic and thermal modules based on the 2D ***H***-formulation and heat conduction equation. The two modules within the FEA environment and their mutual coupling are introduced separately below.

#### 2.2.1. Electromagnetic Module

The electromagnetic behaviors of stacked cables, including AC loss, spatial magnetic field distribution, and current distribution, are obtained by solving the ***H***-formulation [23,24]. The expression of the ***H***-formulation is given in Equation (1):

(1) $$\frac{\partial (\mu_{0}\mu_{r}H)}{\partial t}+\nabla \times \left(\rho \nabla \times H\right)=0$$

where *μ*_0_ and *μ*_r_ are the permeability of free space and the relative permeability of the corresponding material, respectively; *ρ* is the electrical resistivity of the material.

The resistivity of the copper layer, silver layer, and Hastelloy substrate in the cable exhibits temperature dependence [25]. To achieve continuous resistivity during the superconducting transition, a modified resistivity model is adopted to describe the resistivity of the superconducting layer. As shown in Equation (2) [25,26],

(2) $$\rho_{sc}=\left\{\begin{array}{c} \frac{\rho_{s}\cdot \rho_{normal}}{\rho_{s}+\rho_{normal}}\;\;\;\;\;\;\;\;\;\;\;\;\;T<T_{c}\\ \rho_{normal}\;\;\;\;\;\;\;\;\;\;\;\;\;\;\;\;\;\;T\geq T_{c}\; \end{array}\right.$$

where *ρ*_s_ can be expressed by Equation (3) [27,28]. *T*_c_ = 92 K is the superconducting transition temperature of YBCO, and *ρ*_norm_ = 3.5 × 10^−6^ Ω·m is the normal-state resistivity [25].

(3) $$\rho_{s}=\frac{E_{c}}{J_{c}\left(B,T\right)}{\left(\frac{J}{J_{c}\left(B,\;T\right)}\right)}^{n-1}$$

where *E*_c_ = 1 × 10^−4^ V/m, *n* = 32, *J*_c_(***B***, *T*) = *J*_c0_·*J*_c_(***B***)·*J*_c_(*T*) and *J*_c0_ = 2.85 × 10^10^ A/m^2^ is the critical current density under self-field at 77 K [25]. The expressions for *J*_c_(***B***) and *J*_c_(*T*) are given by Equations (4) and (5), respectively [25,29,30,31,32].

(4) $$J_{c}\left(B\right)=\frac{1}{{\left(1+\sqrt{b^{2}{B_{para}}^{2}+{B_{perp}}^{2}}/B_{0}\right)}^{\alpha }}$$

(5) $$J_{c}\left(T\right)=\left\{\begin{array}{c} \frac{T_{c}-T}{T_{c}-T_{0}}\;\;\;\;\;\;\;\;\;\;\;\;\;T_{0}<T<T_{c}\\ 0\;\;\;\;\;\;\;\;\;\;\;\;\;\;\;\;\;\;\;\;\;\;\;\;\;\;\;\;T\geq T_{c}\; \end{array}\right.$$

where *b* = 0.25, *B*_0_ = 52.5 mT, and *α* = 0.7. *T*_0_ = 77 K.

The transport current with the waveform shown in Figure 2 is applied by setting an integral constraint boundary condition. The total loss of the cable, or the total loss of each tape, or the total loss of each component, can be calculated using Equation (6) [16]. The integration period is defined as the last cycle of the alternating current under DC bias:

(6) $$Q_{layer}=\int_{0}^{T}\int qdsdt=\int_{0}^{T}\int E\cdot Jdsdt$$

where the integration domain *s* can be the cross-section of the entire cable, the cross-section of each superconducting tape, or any region of interest, such as the cross-sections of the copper layer, silver layer, or Hastelloy substrate. *T* is the period of the transport current.

#### 2.2.2. Thermal Module

The temperature distribution of the stacked REBCO cable is obtained by solving the heat conduction equation, as shown in Equation (7) [31]:

(7) $$\rho_{m}C_{p}\frac{\partial T}{\partial t}-\nabla \cdot \left(k\nabla T\right)=q$$

where the symbols *ρ*_m_, *C*_p_, and *k* represent the density, specific heat capacity, and thermal conductivity of the material, respectively. These parameters are taken from [25]. *q* is the internal heat source generated by the REBCO cable, as shown in Equation (6). In addition, a convective heat transfer boundary condition is applied to account for thermal exchange between the cable and the surrounding liquid nitrogen, as indicated by the blue line in Figure 1a. The convective heat flux boundary condition can be expressed as shown in Equation (8) [31,33,34]:

(8) $$q_{0}=h(T_{ext}-T)$$

where the convective heat transfer coefficient *h* = 800 W/m^2^/k. The initial temperature of the stacked cable is assumed to be equal to the temperature of the surrounding liquid nitrogen medium, i.e., 77 K.

The coupling between the electromagnetic module and the heat transfer module is realized through *q* and *T*: the loss *q* calculated by the electromagnetic module serves as the heat source term for the heat transfer module, while the temperature *T* obtained from the heat transfer module updates *J*_c_(***B***, *T*) in the electromagnetic module.

## 3. Results and Discussion

### 3.1. Critical Current of REBCO Cables

**Figure 3:**
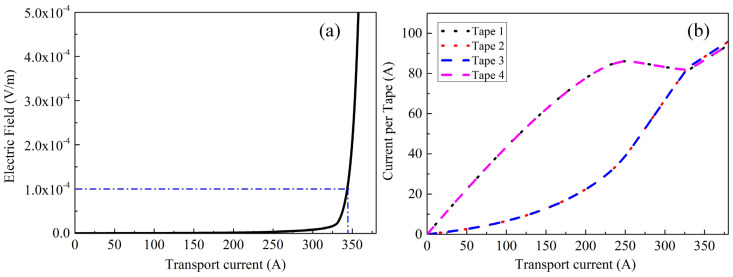
(**a**) *I*–*V* characteristic curve of the stacked cable. (**b**) Variation in the transport current in each superconducting tape with the total transport current of the stacked cable.

A ramp current up to 456 A (i.e., four times the critical current of a single REBCO tape) was applied to the stacked REBCO cable to investigate its critical current characteristics. The simulated *I*–*V* characteristic curve of the cable is shown in Figure 3a. The critical current of the REBCO cable is determined using the average electric field criterion of 1 μV/cm, yielding a critical current of *I*_cable_ = 343.6 A. The critical current of the cable is approximately 75% of the sum of the critical currents of the four independent tapes, which is attributed to the self-field interaction between adjacent tapes inside the cable. The variation in the transport current in each tape with the total cable current is depicted in Figure 3b, where the numbering of each tape is shown in Figure 1a. It can be seen that with the gradual increase in the total transport current of the cable, the outermost two tapes (i.e., Tape 1 and Tape 4) carry a larger current, while the inner two tapes (i.e., Tape 2 and Tape 3) carry a smaller current. Moreover, this difference first increases and then decreases as the total transport current of the cable increases. When the total transport current of the cable reaches approximately 0.95 times its critical current, the transport currents in the four tapes are nearly equal. Afterwards, the transport currents in the inner tapes become slightly larger than those in the outer tapes, which is caused by the difference in local magnetic flux density inside the cable. Compared with the outer tapes, the inner tapes receive a weaker magnetic field contribution from adjacent tapes, which explains why the inner tapes carry a slightly larger proportion of the total current, as described in Ref. [35].

### 3.2. AC Loss of REBCO Cables Without DC Bias

**Figure 4:**
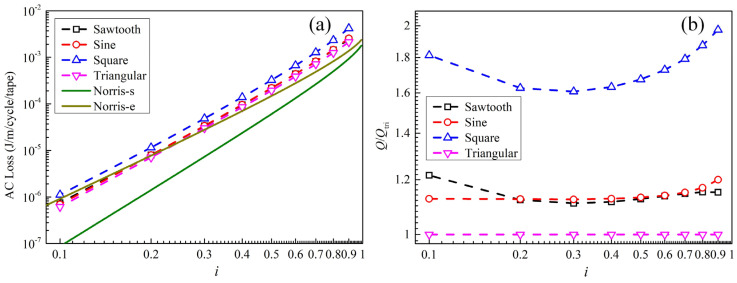
(**a**) Variation characteristics of REBCO cable AC loss with current amplitude under different current waveforms. (**b**) Variation in the ratio of cable AC loss under different current waveform to that under triangular-wave current with transmission current amplitude.

Figure 4a shows the AC loss of the stacked REBCO cable as a function of current amplitude *I*_p_ (*i* = *I*_p_/*I*_cable_) under four different current waveforms. To facilitate a better comparison with the AC loss results for a single tape predicted by the Norris model [36], we divide the AC loss of the stacked cable by four to obtain the average loss per tape in the cable. The results of the Norris model are also plotted in Figure 4a, where Norris-s and Norris-e denote the strip model and elliptical model, respectively. It can be seen that among the four current waveforms, the square wave yields the largest AC loss, while the triangular wave results in the smallest AC loss. This is consistent with the reported conclusions on AC losses of single and stacked tapes under different current waveforms in Refs. [16,17]. The AC losses under the sinusoidal wave and sawtooth wave lie between these two waveforms. Furthermore, the average AC loss per tape in the stacked cable is larger than the loss value predicted by the Norris strip model, which is caused by the mutual superposition of the magnetic fields among the tapes in the stacked cable [17]. To more clearly show the differences in the AC loss of the cable under different current waveforms, Figure 4b plots the ratio of the AC loss under each current waveform to that under the triangular current waveform. It can be seen that the loss generated by the square-wave current is about 1.6–2 times that of the triangular-wave current, while the losses generated by the sawtooth-wave current and sinusoidal-wave current are approximately 1.1–1.2 times that of the triangular-wave current. Therefore, the AC loss of cables varies significantly under different transmission current waveforms.

**Figure 5:**
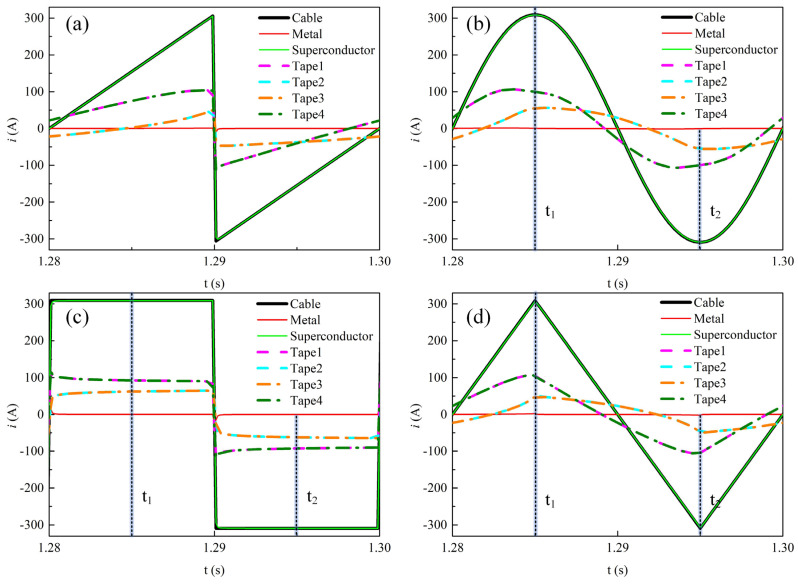
The time-dependent current distribution among different materials and individual tapes in the cable during the final AC cycle when no DC bias is applied and the AC current amplitude is set to 0.9*I*_cable_. (**a**) Sawtooth current, (**b**) sinusoidal current, (**c**) square-wave current, (**d**) triangular current.

Figure 5 shows the time-dependent current waveforms in each component and each REBCO tape within the cable during the last cycle at a transport current amplitude of 0.9*I*_cable_. Overall, during one cycle, the current in the cable is almost entirely confined to the superconducting layer. A small current appears in the metallic layer only at the instants of maximum d*i*/dt for sawtooth and square waveforms. In addition, the transmission current distribution among the stacked tapes in the cable exhibits significant differences. Due to the geometric symmetry of the four stacked tapes, the outer two tapes (Tape 1 and Tape 4) carry identical current, while the inner two tapes (Tape 2 and Tape 3) also carry identical currents. Furthermore, as shown in Figure 5c, under the square-wave current, the amplitude of the current in the outer tapes is larger than that in the inner tapes over one cycle. As shown in Figure 5a,b,d, for the other three waveforms, the current amplitude in the outer tapes is larger than that in the inner tapes during most of the cycle. Such non-uniform current distribution among the tapes results in differences in loss between the tapes.

**Figure 6:**
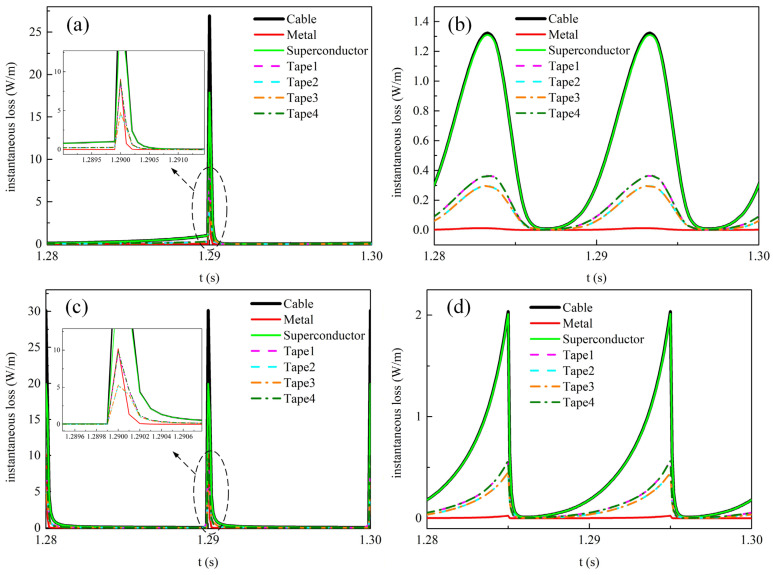
The time-dependent instantaneous loss distribution among different materials and different tapes in the cable during the last cycle when no DC bias is applied and the AC current amplitude is set to 0.9*I*_cable_. (**a**) Sawtooth current, (**b**) sinusoidal current, (**c**) square-wave current, (**d**) triangular current.

Figure 6 depicts the time evolution of instantaneous loss in each component and each tape of the REBCO cable during the last cycle under the four types of transmission current waveforms when the alternating current amplitude is 0.9*I*_cable_. Figure 6a,c show the instantaneous loss distributions under the sawtooth and square-wave transmission currents, respectively. It can be seen that a large instantaneous loss occurs in the cable at the instant when the di/dt reaches its maximum. At this moment, the total loss of the cable comes from both the superconducting layer and the metallic layer, as a distinct peak in the instantaneous loss of the metallic layer can be observed from the locally magnified view. Moreover, calculations reveal that for the sawtooth transmission current, the loss in the metallic layer accounts for 15.3% of the total cable loss, whereas for the square-wave transmission current, the corresponding proportion is 21.4%. This result indicates that the contribution of metallic layer loss to the total loss cannot be neglected when the time rate of change in the transmission current (di/dt) is large. Figure 6b,d show the instantaneous loss under the sinusoidal and triangular-wave transmission currents, respectively. It can be seen that the total loss of the cable is almost entirely contributed by the superconducting layer at any instant. Similarly, by integrating the instantaneous loss over the last cycle, the metallic layer loss accounts for 0.78% of the total cable loss under the sinusoidal transmission current, and 0.82% under the triangular-wave transmission current. Furthermore, for all waveform excitation currents, the transmission loss in the outer tapes of the cable is greater than that in the inner tapes.

**Figure 7:**
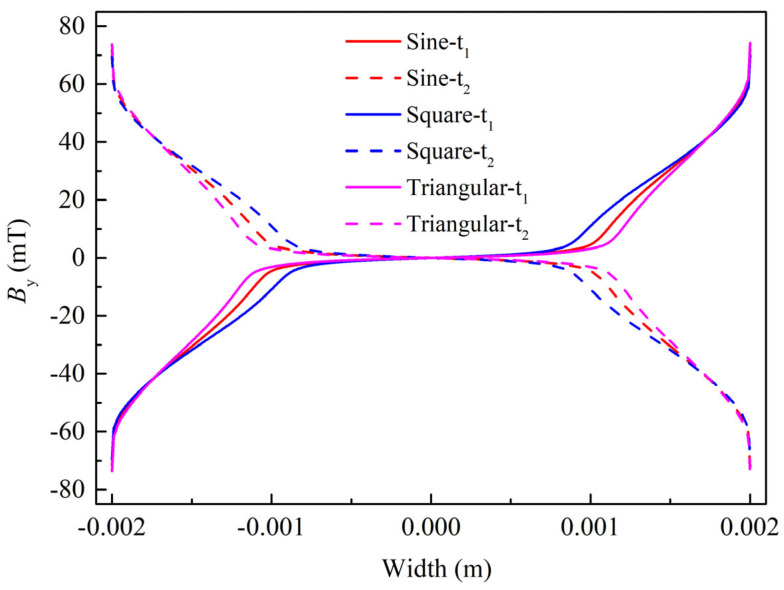
The vertical flux density along the tape width direction in Tape 1 at the peak moment of the last cycle under different transmission current waveforms when no DC bias is applied and the AC current amplitude is set to 0.9*I*_cable_.

Figure 7 shows the distribution of magnetic flux density perpendicular to the tape surface in Tape 1 of the REBCO cable versus the tape width at the peak instant of the last cycle, with the amplitude of the AC current being 0.9*I*_cable_ under different transmission current waveforms. The peak instants t_1_ and t_2_ are indicated by the dashed lines in Figure 5. According to Ref. [16], the flux penetration depth in the tape reaches its maximum at the peak instant of the AC current. The magnitude of the penetration depth is consistent with that of the AC loss in the tape under different waveforms. It can be clearly observed from Figure 7 that the penetration depth is the largest under the square-wave current, followed by the sinusoidal current, and the smallest under the triangular-wave current. This is consistent with the magnitude of AC loss under these three current waveforms. The trend is in agreement with that reported in Ref. [16].

**Figure 8:**
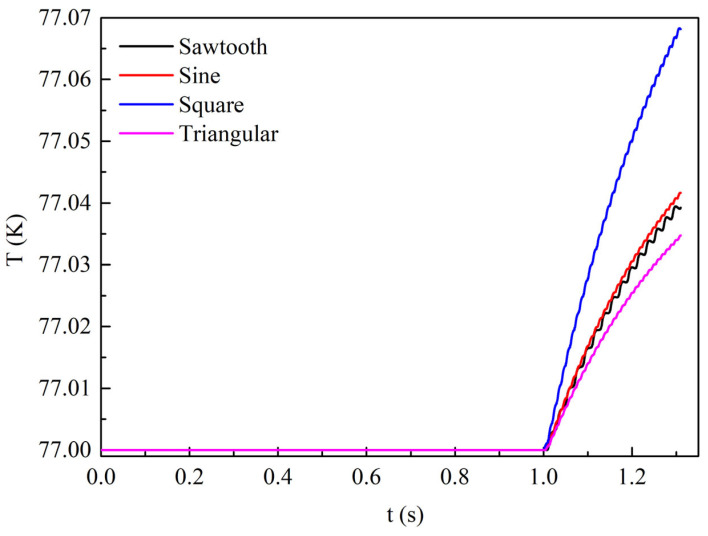
The time variation in the temperature at the center point of the superconducting layer in Tape 1 under different transmission current waveforms when no DC bias is applied and the AC current amplitude is set to 0.9*I*_cable_.

Figure 8 shows the time evolution of the temperature rise at the center of the superconducting layer in Tape 1 of the REBCO cable under four different transmission current waveforms with a current amplitude of 0.9*I*_cable_. From 0 to 1 s, no AC loss is generated since no alternating current is applied, so the tape temperature remains at the ambient temperature of 77 K. From 1 to 1.3 s, AC loss is produced due to the applied alternating current, and the tape temperature gradually increases. The temperature rise is the largest under the square-wave current, the smallest under the triangular-wave current, and similar under the sinusoidal and sawtooth currents, with values lying between those of the square-wave and triangular-wave currents. This trend is consistent with the AC loss under the four current waveforms. Under identical cooling conditions, the temperature rise is determined by the magnitude of the AC loss. Nevertheless, from the perspective of temperature rise magnitude, the temperature rise under the square-wave current is only 0.07 K, which has a negligible effect on the tape performance.

### 3.3. AC Loss of REBCO Cables with DC Bias

**Figure 9:**
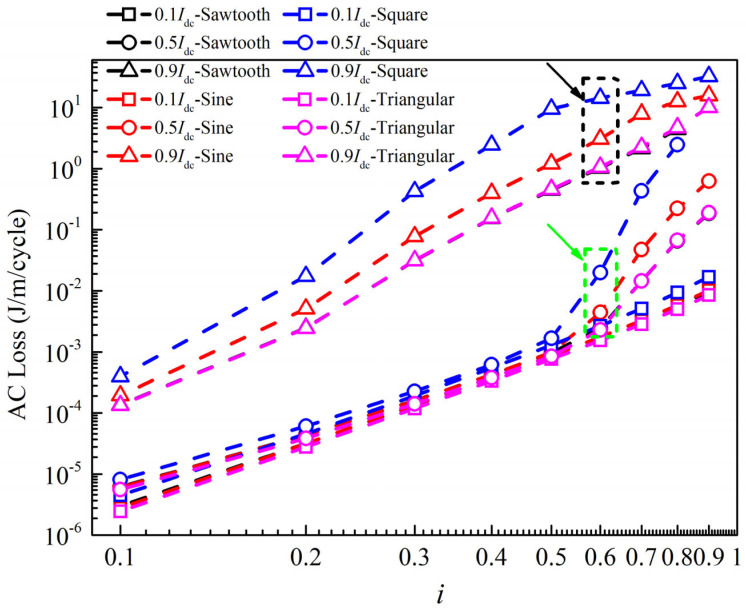
Characteristics of AC loss in cables with the amplitude of alternating current under different current waveforms and DC bias intensities.

Figure 9 shows the characteristics of AC loss in cables as a function of AC current amplitude under different current waveforms and DC bias intensities. In the figure, 0.1*I*_dc_, 0.5*I*_dc_ and 0.9*I*_dc_ represent the DC bias intensities of 0.1*I*_cable_, 0.5*I*_cable_ and 0.9*I*_cable_, respectively. When the DC bias current is small (0.1*I*_dc_), the square-wave current produces the largest loss, while the triangular-wave current produces the smallest loss under alternating currents of different amplitudes. When the DC bias current is moderate (0.5*I*_dc_), the loss of the superconducting cable increases rapidly under the four current waveforms when the AC current amplitude exceeds 0.5*I*_cable_. It can be clearly seen that the loss generated by the square-wave current is the largest, followed by the sinusoidal current, while the triangular-wave and sawtooth-wave currents produce the smallest loss. When the DC bias current is large (0.9*I*_dc_), the loss of the cable under all four current waveforms is significant and increases with the amplitude of the AC current. For example, when the cable carries a triangular-wave current with an amplitude of 0.1*I*_cable_, the AC loss generated at a DC bias current of 0.9*I*_dc_ is 24 times that at a DC bias current of 0.5*I*_dc_. In addition, it can be clearly observed that the square-wave current produces the largest loss, followed by the sinusoidal current, while the triangular-wave and sawtooth-wave currents result in the smallest loss.

**Figure 10:**
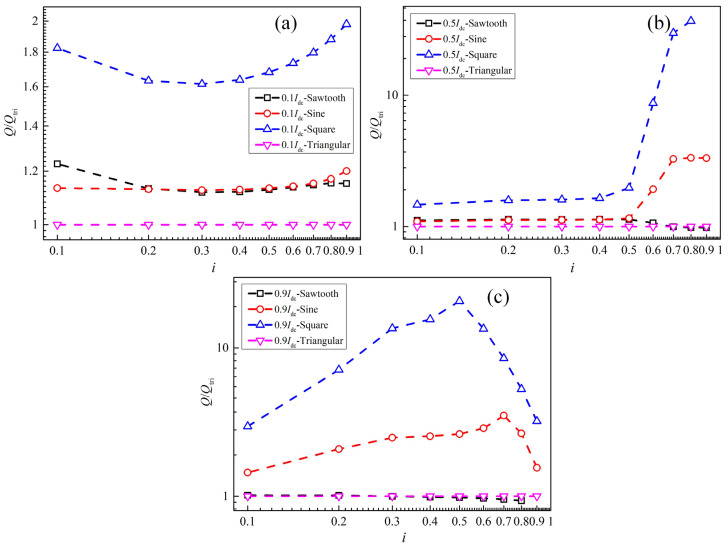
The ratio of the AC loss of the cable under the four alternating current waveforms to that generated by the triangular-wave current at different DC biases: (**a**) 0.1*I*_dc_, (**b**) 0.5*I*_dc_, (**c**) 0.9*I*_dc_.

Figure 10 shows the ratio of the AC loss of the cable under the four alternating current waveforms to that generated by the triangular-wave current at different DC biases. Figure 10a shows the results under the condition of a DC bias current of 0.1*I*_dc_. It can be seen that the loss generated by the square-wave current is the largest, which is about 1.6–2 times that of the triangular-wave current. The losses generated by the sinusoidal and sawtooth currents are approximately 1.1–1.2 times that of the triangular-wave current. As shown in Figure 10b, when the DC bias current is 0.5*I*_dc_ and the amplitude of the alternating current is greater than 0.5*I*_cable_, the losses generated by the square-wave and sinusoidal currents are significantly larger than those generated by the sawtooth and triangular currents. For example, when the AC current amplitude is 0.6*I*_cable_, the loss generated by the square-wave current is 8.7 times that produced by the triangular-wave current, and the loss generated by the sinusoidal current is 1.9 times that produced by the triangular-wave current. When the DC bias current is 0.9*I*_dc_, the loss generated by the square-wave current is greater than that by the sinusoidal current, while the losses produced by the sawtooth current and triangular current are approximately the same and the smallest.

**Figure 11:**
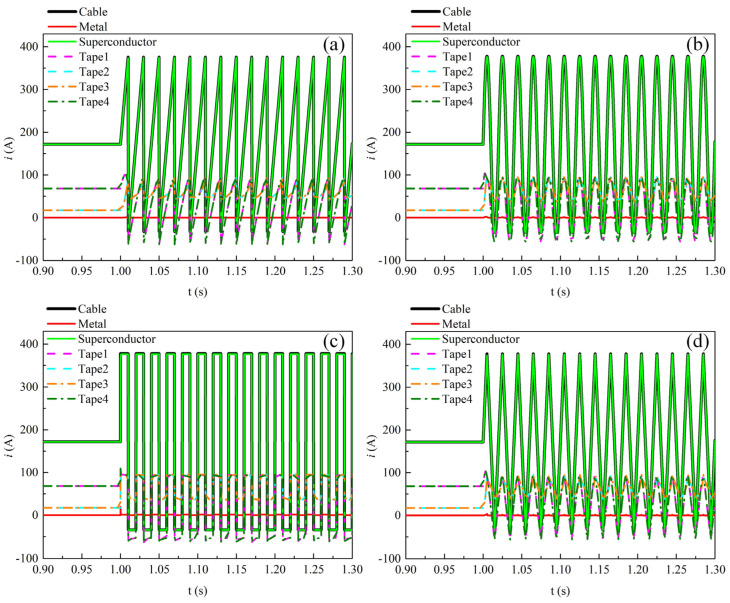
The temporal variations in transport current in each component and each tape of the REBCO cable at a DC bias current of 0.5*I*_dc_ and an AC current amplitude of 0.6*I*_cable_. (**a**) Sawtooth current, (**b**) sinusoidal current, (**c**) square-wave current, (**d**) triangular current.

Figure 11 shows the temporal variations in the transport current in each component and each tape of the REBCO cable at a DC bias current of 0.5*I*_dc_ and an AC current amplitude of 0.6*I*_cable_, i.e., the operating parameters corresponding to the loss values indicated by the green dashed box in Figure 9. Overall, under the four current waveforms, the time-varying transport current is almost entirely distributed in the superconducting layer of the cable. There is no gradual increase over time in the current shunting toward the metallic layer. This indicates that the temperature rise in the cable is small under this operating condition, which will be further illustrated in the subsequent text. During one cycle of the alternating current variation, the variation amplitude of the transport current in the outer tapes (Tape 1 and Tape 4) is larger than that in the inner tapes (Tape 2 and Tape 3). However, at the instant when the transport current reaches its positive peak, the transport currents of the four tapes are nearly equal. This is because the total current carried by the cable reaches 1.1*I*_cable_ at this moment, and all four tapes are fully penetrated.

**Figure 12:**
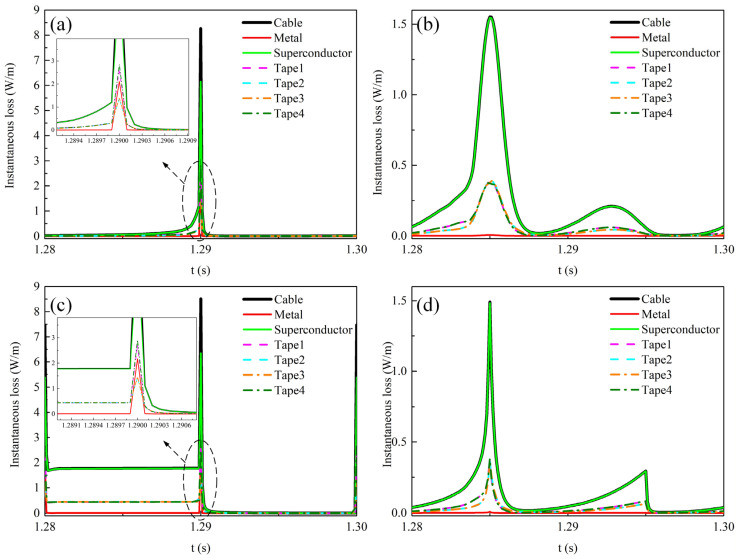
The temporal variations in instantaneous loss in each component and each tape of the REBCO cable during the last cycle at a DC bias current of 0.5*I*_dc_ and an AC current amplitude of 0.6*I*_cable_. (**a**) Sawtooth current, (**b**) sinusoidal current, (**c**) square-wave current, (**d**) triangular current.

Figure 12 shows the temporal variations in instantaneous loss in each component and each tape of the REBCO cable during the last cycle at a DC bias current of 0.5*I*_dc_ and an AC current amplitude of 0.6*I*_cable_. Figure 12a,c show the instantaneous loss distributions under the sawtooth and square-wave currents, respectively. Similarly, the instantaneous loss of the cable reaches its peak when the time rate of current change d*i*/dt is maximized, at which point the instantaneous loss in the metallic layer also exhibits a peak. The reason is that a strong electric field is induced in the metallic layer at this moment, thereby generating significant induced eddy currents. The eddy current loss increases sharply, leading to a peak loss in the metallic layer. Furthermore, when the transport alternating current is the square-wave current, the cable loss is non-zero during the first half period of the instantaneous loss. This is because the transport current amplitude reaches 1.1*I*_cable_, exceeding the cable’s critical current. In the mixed state of superconductors, this leads to flux flow driven by the DC bias, which introduces resistivity and consequently increases the AC loss in the cable [7]. In addition, under the four current waveforms, the loss in the first half-cycle is larger than that in the second half-cycle, because the amplitude of the positive transport current is greater in the first half-cycle.

**Figure 13:**
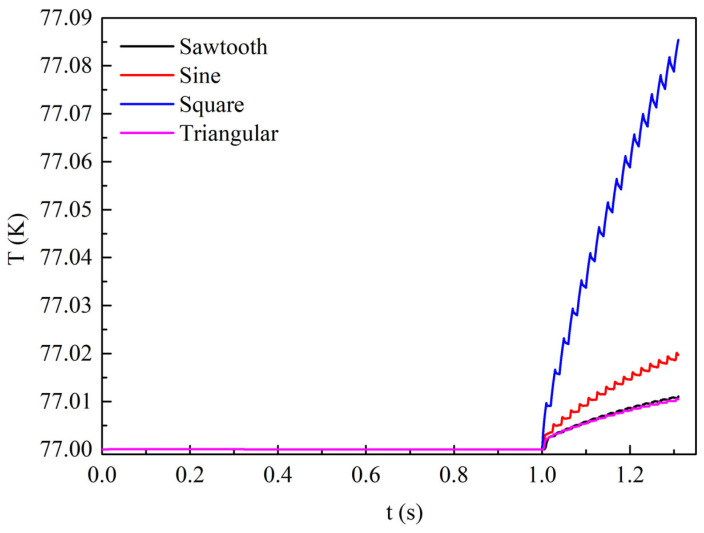
The temperature variation at the center of the superconducting layer in Tape 1 of the REBCO cable over time under four transmission current waveforms, with a DC bias current of 0.5*I*_dc_ and an AC current amplitude of 0.6*I*_cable_.

Figure 13 shows the temperature variation at the center of the superconducting layer of Tape 1 in the REBCO cable over time under a DC bias current of 0.5*I*_dc_ and an AC current amplitude of 0.6*I*_cable_. Similarly, it can be observed that the temperature rise variation under the four current waveforms is consistent with that of the loss. Specifically, the temperature rise is the largest for the square-wave current, followed by the sinusoidal current, while the temperature rises for the sawtooth and triangular-wave currents are nearly identical and the lowest. Under this operating condition, the temperature rise in the tapes is also very low. For example, the temperature rise under the square-wave current is only approximately 0.09 K.

**Figure 14:**
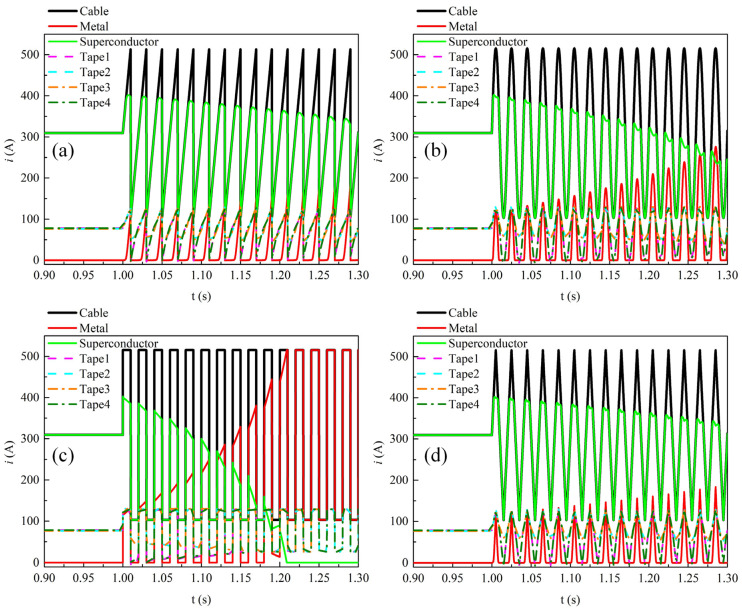
The temporal variations in transport current in each component and each tape of the REBCO cable at a DC bias current of 0.9*I*_dc_ and an AC current amplitude of 0.6*I*_cable_. (**a**) Sawtooth current, (**b**) sinusoidal current, (**c**) square-wave current, (**d**) triangular current.

Figure 14 shows the temporal variations in the transport current in each component and each tape of the REBCO cable at a DC bias current of 0.9*I*_dc_ and an AC current amplitude of 0.6*I*_cable_, i.e., the operating parameters corresponding to the loss values indicated by the black dashed box in Figure 9. Figure 14a,b,d show the time variation in the cable current under the sawtooth, sinusoidal, and triangular-wave transport currents, respectively. It can be seen that as time increases, the peak current in the superconducting layer of the cable gradually decreases, while the peak current in the metallic layer gradually increases. This is because the temperature rise reduces the critical current of the tapes. Figure 14c shows the time variation in the cable current under the square-wave transport current. When the time reaches 1.209 s, the transport current in the superconducting layer approaches 0 A, and the transport current in the cable is thereafter only distributed in the metallic layer, which is caused by the quenching of the superconducting layer.

**Figure 15:**
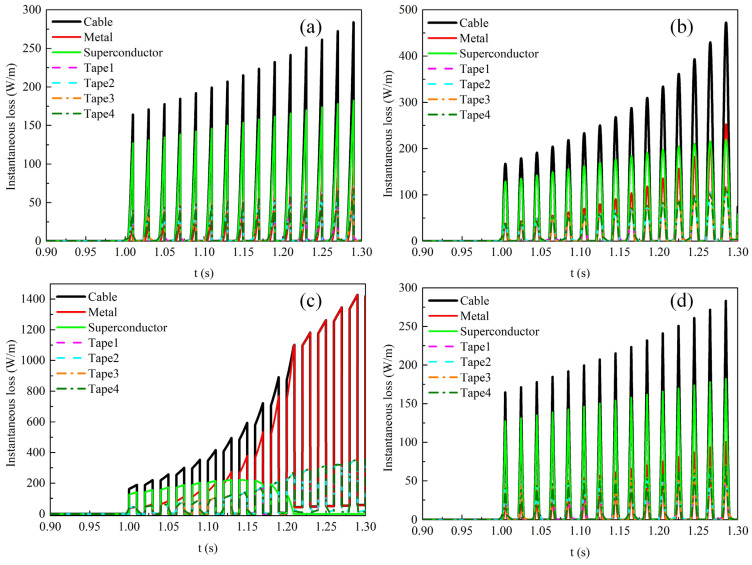
The temporal variations in instantaneous loss in each component and each tape of the REBCO cable at a DC bias current of 0.9*I*_dc_ and an AC current amplitude of 0.6*I*_cable_. (**a**) Sawtooth current, (**b**) sinusoidal current, (**c**) square-wave current, (**d**) triangular current.

Figure 15 shows the temporal variations in instantaneous loss in each component and each tape of the REBCO cable at a DC bias current of 0.9*I*_dc_ and an AC current amplitude of 0.6*I*_cable_. Figure 15a,b,d show the time variation in the instantaneous loss in the cable under the sawtooth, sinusoidal, and triangular-wave currents, respectively. Under these three current waveforms, the cable loss increases gradually over time, and both the superconducting layer loss and metallic layer loss also increase monotonically. This is due to the temperature rise, which causes the total cable current to gradually shunt into the metallic layer. Figure 15c shows the time variation in the instantaneous loss in the cable under the square-wave transport current. At 1.209 s, the loss in the superconducting layer drops to 0 W/m, and the total cable loss thereafter comes entirely from the metallic layer.

**Figure 16:**
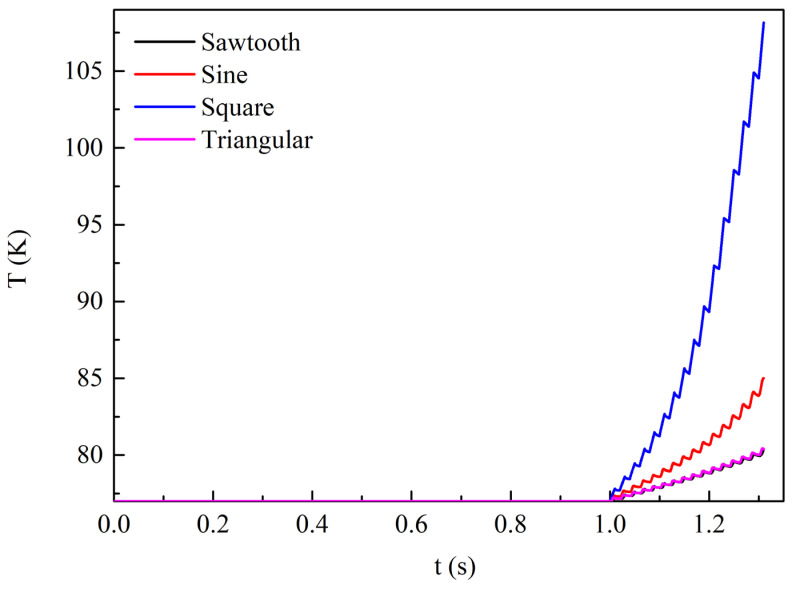
The temperature variation at the center of the superconducting layer in Tape 1 of the REBCO cable over time under four transmission current waveforms, with a DC bias current of 0.9*I*_dc_ and an AC current amplitude of 0.6*I*_cable_.

Figure 16 shows the temperature variation at the center of the superconducting layer of Tape 1 in the REBCO cable over time under a DC bias current of 0.9*I*_dc_ and an AC current amplitude of 0.6*I*_cable_. At this time, the temperature rise in the tapes is significant under all four current waveforms. Within the simulated time range, the REBCO superconducting cable quenches at 1.209 s under the square-wave current, with a maximum temperature of 108 K. Next, the maximum temperature under the sinusoidal current reaches 85 K, while the maximum temperatures under the sawtooth and triangular-wave currents are nearly equal at 80 K. Similarly, the temperature rise variation under the four alternating currents is consistent with the loss variation.

These results indicate that the AC loss of the cable increases significantly when the peak current, defined as the sum of the DC bias current and the AC amplitude, exceeds the cable’s critical current. At relatively high DC bias currents, the multilayer electromagnetic–thermal coupled model can more adequately describe the loss, current sharing, and temperature rise behaviors of the cable.

## 4. Conclusions

In this paper, the electromagnetic–thermal characteristics of REBCO cables excited by four typical current waveforms under DC bias are investigated based on the ***H***-formulation and heat conduction equation. The DC bias current ranges from 0.1 to 0.9 times the critical current of the cable, and the peak value of the AC current varies from 0 to 0.9 times the critical current of the cable. In the absence of DC bias, the square-wave current generates the largest loss, while the triangular-wave current produces the smallest loss. The losses caused by the sinusoidal current and the sawtooth current are similar and lie between the above two waveforms. When the DC bias current is small, e.g., 0.1*I*_dc_, the loss characteristics of the four current waveforms are consistent with those under zero DC bias. When the DC bias current is 0.5*I*_dc_ and the amplitude of the alternating current is greater than 0.5*I*_cable_, the loss of the cable under the four current waveforms increases rapidly. The loss is the largest under the square-wave current, followed by the sinusoidal current, while the losses generated by the sawtooth current and the triangular current are comparable. When the DC bias current is high, e.g., 0.9*I*_dc_, even a small amplitude alternating current for the four waveforms can result in high losses. For example, when the cable carries a triangular-wave current with an amplitude of 0.1*I*_cable_, the AC loss generated at a DC bias current of 0.9*I*_dc_ is 24 times that at a DC bias current of 0.5*I*_dc_.

## Figures and Tables

**Table 1:** The specifications of the HTS tape and cable.

Parameters	Value
Copper layer width	4.04 mm
Copper layer thickness	20 μm
Silver layer width	4 mm
Silver layer thickness	2 μm
Superconducting layer width	4 mm
Superconducting layer thickness	1 μm
Substrate layer width	4 mm
Substrate layer thickness	50 μm
Cable width	4.04 mm
Cable thickness	0.38 mm

## Data Availability

The original contributions presented in this study are included in the article. Further inquiries can be directed to the corresponding authors.
